# Diagnostic value and relative weight of sequence-specific magnetic resonance features in characterizing clinically significant prostate cancers

**DOI:** 10.1371/journal.pone.0178901

**Published:** 2017-06-09

**Authors:** Olivier Rouvière, Tristan Dagonneau, Fanny Cros, Flavie Bratan, Laurent Roche, Florence Mège-Lechevallier, Alain Ruffion, Sébastien Crouzet, Marc Colombel, Muriel Rabilloud

**Affiliations:** 1 Hospices Civils de Lyon, Department of Urinary and Vascular Imaging, Hôpital Edouard Herriot, Lyon, France; 2 Université de Lyon, Lyon, France; Université Lyon 1, faculté de médecine Lyon Est, Lyon, France; 3 Inserm, U1032, LabTau, Lyon, France; 4 Hospices Civils de Lyon, Service de Biostatistique et Bioinformatique, Lyon, France; CNRS, UMR5558, Laboratoire de Biométrie et Biologie Evolutive, Equipe Biotatistique-Santé, Villeurbanne, France; 5 Hospices Civils de Lyon, Department of Pathology, Hôpital Edouard Herriot, Lyon, France; 6 Hospices Civils de Lyon, Department of Urology, Centre Hospitalier Lyon Sud, Pierre Bénite, France; 7 Hospices Civils de Lyon, Department of Urology, Hôpital Edouard Herriot, Lyon, France; Taipei Medical University, TAIWAN

## Abstract

**Purpose:**

To assess the diagnostic weight of sequence-specific magnetic resonance features in characterizing clinically significant prostate cancers (csPCa).

**Materials and methods:**

We used a prospective database of 262 patients who underwent T2-weighted, diffusion-weighted, and dynamic contrast-enhanced (DCE) imaging before prostatectomy. For each lesion, two independent readers (R1, R2) prospectively defined nine features: shape, volume (V_Max), signal abnormality on each pulse sequence, number of pulse sequences with a marked (S_Max) and non-visible (S_Min) abnormality, likelihood of extracapsular extension (ECE) and PSA density (dPSA). Overall likelihood of malignancy was assessed using a 5-level Likert score. Features were evaluated using the area under the receiver operating characteristic curve (AUC). csPCa was defined as Gleason ≥7 cancer (csPCa-A), Gleason ≥7(4+3) cancer (csPCa-B) or Gleason ≥7 cancer with histological extraprostatic extension (csPCa-C),

**Results:**

For csPCa-A, the Signal1 model (S_Max+S_Min) provided the best combination of signal-related variables, for both readers. The performance was improved by adding V_Max, ECE and/or dPSA, but not shape. All models performed better with DCE findings than without.

When moving from csPCa-A to csPCa-B and csPCa-C definitions, the added value of V_Max, dPSA and ECE increased as compared to signal-related variables, and the added value of DCE decreased.

For R1, the best models were Signal1+ECE+dPSA (AUC = 0,805 [95%CI:0,757–0,866]), Signal1+V_Max+dPSA (AUC = 0.823 [95%CI:0.760–0.893]) and Signal1+ECE+dPSA [AUC = 0.840 (95%CI:0.774–0.907)] for csPCa-A, csPCA-B and csPCA-C respectively. The AUCs of the corresponding Likert scores were 0.844 [95%CI:0.806–0.877, p = 0.11], 0.841 [95%CI:0.799–0.876, p = 0.52]) and 0.849 [95%CI:0.811–0.884, p = 0.49], respectively.

For R2, the best models were Signal1+V_Max+dPSA (AUC = 0,790 [95%CI:0,731–0,857]), Signal1+V_Max (AUC = 0.813 [95%CI:0.746–0.882]) and Signal1+ECE+V_Max (AUC = 0.843 [95%CI: 0.781–0.907]) for csPCa-A, csPCA-B and csPCA-C respectively. The AUCs of the corresponding Likert scores were 0. 829 [95%CI:0.791–0.868, p = 0.13], 0.790 [95%CI:0.742–0.841, p = 0.12]) and 0.808 [95%CI:0.764–0.845, p = 0.006]), respectively.

**Conclusion:**

Combination of simple variables can match the Likert score’s results. The optimal combination depends on the definition of csPCa.

## Introduction

Multiparametric Magnetic Resonance (MR) imaging can detect clinically significant prostate cancer (csPCa) with good accuracy [1–7]. Unfortunately, its interpretation needs expertise. Indeed, prostate focal lesions can have very different appearances from one MR pulse sequence to another, and it may be difficult to distinguish, within the large number of combinations of shape and signal abnormalities, those that are benign from those that are malignant.

Because it is impossible to definitely characterize as benign or malignant all prostate focal lesions, the use of a 5-point subjective score has been widely encouraged to describe the level of suspicion of a given lesion [8, 9]. This so-called Likert score is a highly significant predictor of the malignant nature of prostate focal lesions [3, 10–13]. However, because there are no descriptions of specific criteria to be used in the scoring process, the Likert score relies heavily on the reader’s experience. Therefore, some research groups tried to set up more objective scoring systems to improve inter-reader agreement [14–17].

In 2012, the European Society of Urogenital Radiology (ESUR) endorsed the Prostate Imaging Reporting and Data System (PIRADS) score [18]. It was shown to be a significant predictor of malignancy and aggressive behavior [19, 20]. However, it did not outperform the Likert score, at least for experienced readers, and did not improve inter-reader agreement either [11, 21, 22]. Two main limitations were identified. First, it gave the same diagnostic weight to all pulse sequences, and second the interpretation of dynamic contrast-enhanced (DCE) imaging relied mostly on the shape of the enhancement curve that was shown to be a poor predictor of malignancy [11].

In 2015, the ESUR and the American College of Radiology endorsed the so-called PIRADS v2 score [23, 24] that introduced the concept of a dominant pulse sequence bearing most of the diagnostic weight (diffusion-weighted [DW] imaging for the peripheral zone [PZ]; T2-weighted [T2W] imaging for the transition zone [TZ]) and that limited the role of DCE imaging. This PIRADS v2 score gave good results in characterizing prostate focal lesions [25, 26], but, again, some limitations were pointed out [27]. It does not seem to improve inter-reader agreement as compared to the PIRADS v1 score, even after training [28, 29], results in a high false positive rate [30] and was outperformed by alternative in-house scores [17, 28]. These results have led some authors to suggest that there might be structural limits to the ability of any score based on MR imaging to allow detection of prostate cancer with high specificity [28].

The PIRADS scores were not based on an analysis of a large dataset, but rather were a result of expert opinion. As others [17], we hypothesize that processing large prospective databases detailing sequence-specific findings may help further refining prostate MR scoring systems. We therefore undertook this study to assess the relative diagnostic weight of sequence-specific MR features in characterizing aggressive cancers in PZ, using a prospectively acquired radiologic-pathologic database of patients treated by prostatectomy.

## Material and methods

### Radiologic-pathologic database

As of September 2008, all patients who underwent prostate multiparametric MR imaging before radical prostatectomy at our institution (Hospices Civils de Lyon) were proposed to have their radiologic and pathologic data entered in a prospective database approved by our Institutional Review Board (Comité de Protection des Personnes Sud-Est IV). All patients gave written informed consent. The database was used for other studies evaluating prostate cancer detection rates [3] and accuracy of tumor volume estimation [31] at multiparametric MR imaging, existing MR scoring systems [11] and diagnostic accuracy of quantitative MR parameters [32–34]. These studies do not overlap with the present one that aims at understanding the relative weight of non-quantitative sequence-specific findings in the characterization of aggressive prostate cancers, in order to improve current MR scoring systems.

### MR image analysis

MR examinations comprised at least T2W, DW and DCE imaging at 1.5T and 3T, but protocol parameters varied as per the standard of care at the time of the examination (S1 Table). Upon inclusion, preoperative MR examinations were prospectively analyzed by two senior readers blinded to clinical and histological data. Reader 1 (R1, OR) and 2 (R2, FB) had respectively 11 years and 1 year of experience at the start of the database in 2008. Readers independently described all prostate suspicious focal lesions. They took into account all PZ lesions showing low-signal intensity on T2W images and/or on apparent diffusion coefficient (ADC) maps, and/or showing early enhancement at visual inspection of DCE images.

First, readers delineated each lesion on all three MR pulse sequence images using the Osirix software (Osirix imaging software, Geneva, Switzerland). This allowed the calculation of the lesion volume on each MR pulse sequence.

Second, readers specified the lesion shape on the three MR pulse sequences images, using the following list: not visible; ill-defined area with indistinct margins; linear lesion perpendicular to the capsule; linear lesion parallel to the capsule; triangular lesion; nodular lesion without mass effect on the adjacent TZ or capsule; nodular lesion with mass effect on the adjacent TZ or capsule.

Third, they separately noted the degree of signal abnormality on T2W, DW and DCE images using the following qualitative 4-level scale: 0, not visible; 1, mild abnormality; 2, moderate abnormality; 3, marked abnormality.

Fourth, they assessed the likelihood of extracapsular extension (ECE) using a subjective 5-level score: 1, definitely absent; 2, likely absent; 3, indeterminate; 4, likely present; 5, definitively present.

Finally, they assessed the likelihood of malignancy of each lesion, using the following subjective 5-level Likert score: 1, definitely benign; 2, likely benign; 3, indeterminate; 4, likely malignant; 5, definitely malignant. Because a Likert score of 1/5 was used only in areas with normal appearance on all pulse sequences, focal lesions had, by definition, a Likert score ≥2/5.

### Comparison of MR and histopathologic findings

Whole-mount sections of prostatectomy specimens were obtained every 3 mm according to guidelines [35]. A single uropathologist (FML) with 10 years of experience at the start of the database and blinded to the readers’ assessements, assigned individual Gleason scores to all cancer foci and delineated them on the glass cover of whole-mount sections. Then, the readers and the uropathologist compared MR and histopathologic findings. The pathologist decided which MR lesions matched the positions of histologic cancers. These matching lesions were considered true positives only if their largest diameter was 50–150% of the diameter of the corresponding cancer to minimize chance detection [36, 37].

### Assessment of variables and combination of variables in characterizing csPCa-A in PZ

We first assessed the performance of the 9 variables decribed in Table 1 in characterizing in PZ csPCa defined as Gleason ≥7 cancers, i.e. as cancers with an International Society of Urological Pathology (ISUP) grade group ≥2 (csPCa-A) [38]. The variables comprised the PSA density (dPSA) and eight variables describing MR lesions (S_T2, S_DW, S_DCE, S_Max, S_Min, Shape, ECE and Vmax).

**Table 1:** Assessed individual variables.

	Description	Possible values
S_T2	Degree of signal abnormality on T2W	not visible, mild, moderate, marked
S_DW	Degree of signal abnormality on DW	not visible, mild, moderate, marked
S_DCE	Degree of signal abnormality on DCE	not visible, mild, moderate, marked
S_Max	Number of MR pulse sequences showing a marked signal abnormality	0, 1, 2, 3
S_Min	Number of MR pulse sequences on which the lesion was not visible	0, ≥1
Shape	Shape of the lesion on T2W ^(1)^	not visible, ill-defined area, linear perpendicular to the capsule, linear parallel to the capsule, triangular, nodular without mass effect, nodular with mass effect ^(2)^
ECE	Extracapsular extension score	1/5, 2/5, 3/5, 4/5 or 5/5
V_Max	Largest of the lesion’s volumes measured on the three MR pulse sequences (mL)	Continuous variable
dPSA	PSA density (ng/mL/mL)	Continuous variable

T2W: T2-weighted imaging; DW: diffusion-weighted imaging; DCE: dynamic contrast-enhanced imaging

(1) If the lesion was not visible on T2W images, the shape on DW images was taken into consideration. If the lesion was not visible also on DW images, DCE images were used to describe the shape of the lesion.

(2) Ill-defined areas and linear lesions perpendicular to the capsule were grouped in a single category for statistical analysis.

Then we assessed the performance of three combinations of variables related to the lesions’ signal. Signal1 model comprised S_Max and S_ Min, Signal2 model comprised S_Max, S_ Min and S_DW, and Signal3 model comprised S_T2, S_DW, S_DCE. Signal1a, Signal2a and Signal3a models used the three MR pulse sequences. Signal1b, Signal2b and Signal3b models did not use DCE imaging. In Signal2a model, S_Max and S_Min used only the results of T2W and DCE imaging since DW findings were coded by S_DW. In Signal2b model, S_Max and S_Min used only the results of T2W imaging. These models were compared to S_Max as a stand-alone, with (S_Maxa) and without (S_Maxb) use of DCE imaging.

Signal1 model was selected for the rest of the analysis. The variables Shape, ECE, Vmax and dPSA were sequentially added to it. Each resulting multivariable model was assessed first using the findings of the three MR pulse sequences and then without DCE imaging.

### Alternative definitions of csPCa in PZ

The 9 individual variables, the Signal1, Signal2 and Signal3 models, and the combinations of the Signal1 model and the variables Shape, ECE, Vmax and dPSA were also assessed using two alternative definitions for csPCa: (i) csPCa-B: Gleason ≥7(4+3) (ISUP grade group ≥3) and (ii) csPCa-C: Gleason ≥7 (ISUP Grade group ≥2) and histologically-proven extraprostatic extension (pathological stage ≥pT3a).

In the database, extraprostatic extension was assessed at the lesion level (i.e., it was specified whether each lesion showed features of extraprostatic extension at pathological examination). This allowed combining the Gleason score and extraprostatic extension features at the lesion level.

### Statistical analysis

The analysis units were the lesions identified by each reader. The probability that a lesion corresponded to a csPCa was modelled using a logistic regression for each of the 9 studied variables, and for the different combinations of variables described above. Receiver operating characteristic (ROC) curves were built using the probabilities of csPCa predicted by the different models. The diagnostic performance of each variable and combination of variables was quantified using the area under the ROC curve (AUC). Because the same data were used for developing the models and assessing their performance, the AUC can be overestimated. This is often referred to as optimism. We therefore used a bootstrap procedure to estimate a corrected AUC as proposed by Harrel [39] (S1 Appendix). To take account of the clustered structure of the data, the bootstrap procedures used a clustered resampling at the patient level. A similar bootstrap procedure was used for model-to-model comparison and for constructing confidence intervals. All interval estimations given in this paper are 95% confidence intervals (95%CI). Analyses were performed using R software version 3.2.4 (http://cran.r-project.org).

## Results

### Study population

At the time of analysis, the database contained 262 patients imaged between September 2008 and February 2013. MR imaging was performed at 1.5T on scanner A (n = 72) or at 3T on scanner B (n = 113) or C (n = 77). The patients median age, PSA level and PSA density at the time of imaging were 62 years (interquartile range (IQR), 58–66), 6.5 ng/mL (IQR, 5.0–9.9) and 0.16 ng/mL/mL (IQR, 0.12–0.25) respectively.

**Fig 1:**
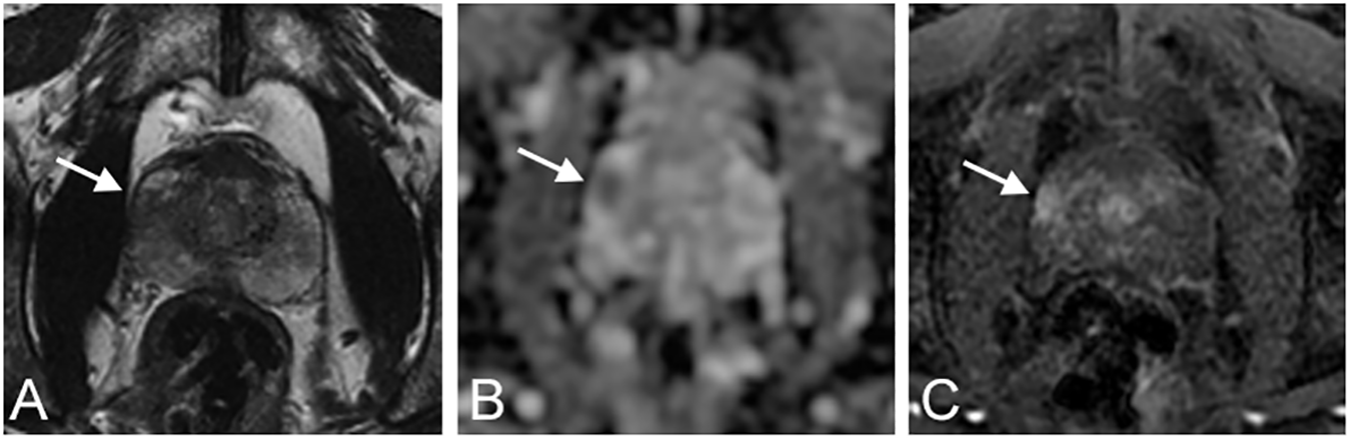
Axial multiparametric MR images acquired on scanner B at 3T, in a 58 year-old patient with a PSA density of 0.28 ng/mL/mL. A) T2-weighted image. B) Apparent diffusion coefficient map. C) Dynamic contrast-enhanced image. One suspicious lesion was described by both readers in the right peripheral zone (A-C, arrow). The lesion was noted as nodular without mass effect by both readers. S_T2, S_DW and S_DCE were respectively marked, marked and moderate for both readers. V_Max was 2.0 cc and 2.1 cc for readers 1 and 2 respectively. The ECE and Likert scores were respectively 2/5 and 5/5 for both readers. Analysis of the prostatectomy specimen showed a matching Gleason 9 (4+5) cancer with a histological volume of 1.6 cc.

R1 described 474 PZ lesions in 250 patients. Two lesions were excluded because of artefacts on DW imaging, leaving 472 lesions (204 Gleason ≥7 cancers, 79 Gleason ≤6 cancers and 189 benign findings). R2 described 392 PZ lesions in 242 patients. Three lesions were excluded because of artefacts on DW imaging leaving 389 lesions (191 Gleason ≥7 cancers, 64 Gleason ≤6 cancers and 134 benign findings; Fig 1). In total, 21.4% (101/472) and 26% (101/389) of the lesions described by R1 and R2 respectively showed histologically-proven extracapsular extension.

The percentage of csPCa-A was 43.2% (204/472) for R1 and 48.7% (191/389) for R2. The percentage of csPCa-B was 21.8% (103/471) for R1 and 25.8% (101/392) for R2. The percentage of csPCa-C was 20.8% (98/471) for R1 and 24.5% (96/392) for R2.

### Assessment of individual variables in characterizing csPCa-A in PZ

S2 Table shows the distribution of individual variables for tissue classes and readers. S_Max obtained the highest AUC for both readers (Table 2). For R1, it was significantly different from that of S_T2 (p = 0.002), S_DW (p = 0.01) and S_DCE (p = 0.005). For R2, it was significantly different from that of S_T2 (p = 0.001) and S_DCE (p = 0.011) but not from that of S_DW (p = 0.094)

**Table 2:** Performance of individual variables in characterizing csPCa-A in PZ.

	AUC (95%CI)	
	Reader 1	Reader 2
S_T2	0.709 (0.647–0.769)	0.631 (0.567–0.700)
S_DW	0.731 (0.672–0.793)	0.701 (0.639–0.769)
S_DCE	0.686 (0.630–0.751)	0.675 (0.600–0.745)
S_Max	0.785 (0.727–0.839)	0.759 (0.688–0.829)
S_Min	0.604 (0.555–0.652)	0.566 (0.53–0.603)
Shape	0.654 (0.594–0.724)	0.667 (0.589–0.745)
ECE	0.735 (0.685–0.788)	0.702 (0.634–0.77)
V_Max	0.649 (0.579–0.718)	0.654 (0.577–0.732)
dPSA	0.581 (0.473–0.649)	0.603 (0.498–0.68)

AUC: area under the receiver operating characteristic curve with optimism correction; 95%CI: 95% confidence intervals

### Assessment of signal-related models in characterizing csPCa-A in PZ

Table 3 shows the results of S_Maxa-b, Signal1a-b, Signal2a-b and Signal3a-b models. For both readers, all models performed better with use of DCE imaging, and the difference was statistically significant for S_Max and Signal1 models for R1. As compared to S_Max, the Signal1-3 models either performed worse or yielded only marginal improvement. The highest AUC was obtained by Signal1a model for both readers. However the AUC differences (ΔAUC) with S_Maxa were only +0.009 for R1 and +0.010 for R2, and not statistically significant (p = 0.49 for R1 and p = 0.14 for R2).

**Table 3:** Performance of models based on combinations of signal abnormalities in characterizing csPCa-A in PZ.

	Description	Reader 1			Reader 2		
		AUC (95%CI)		P ^(1)^	AUC (95%CI)		P^(1)^
		With DCE	Without DCE		With DCE	Without DCE	
S_Max		0.785 (0.727–0.839)	0.742 (0.681–0.801)	0.015	0.759 (0.688–0.829)	0.725 (0.654–0.789)	0.107
Signal1	S_Max + S_Min	0.794 (0.734–0.851)	0.748 (0.683–0.808)	0.015	0.769 (0.698–0.835)	0.725 (0.658–0.791)	0.060
Signal2	S_Max + S_Min + S_DW	0.750 (0.700–0.822)	0.719 (0.657–0.790)	0.057	0.760 (0.687–0.830)	0.703 (0.637–0.771)	0.061
Signal3	S_T2 + S_DW + S_DCE	0.784 (0.736–0.848)	0.757 (0.695–0.821)	0.051	0.762 (0.702–0.840)	0.720 (0.656–0.794)	0.088

DCE: dynamic contrast-enhanced imaging; AUC: area under the receiver operating characteristic curve with optimism correction; 95%CI: 95% confidence intervals

(1) P value comparing the performances of the model with and without DCE imaging.

### Assessment of other multivariable models in characterizing csPCa-A in PZ

Table 4 shows the results of the models associating Signal1 model and other variables. When DCE imaging was used, the addition of other variables either decreased the diagnostic performance or slightly improved it, with a maximal ΔAUC of +0.011 for R1 and +0.021 for R2. When DCE imaging was not used, almost all multivariable models performed better than Signal1b model with a maximum ΔAUC of +0.037 for R1 and +0.040 for R2.

**Table 4:** Performances of multivariable models in characterizing csPCa-A in PZ.

	AUC (95%CI)–Reader 1		AUC (95%CI)–Reader 2	
	With DCE	Without DCE	With DCE	Without DCE
**Signal1**	0.794 (0.734–0.851)	0.748 (0.683–0.808)	0.769 (0.698–0.835)	0.725 (0.658–0.791)
**Signal1 + Shape**	0.784 (0,729–0,848)	0.750 (0.687–0.818)	0.762 (0.705–0.837)	0.723 (0.666–0.806)
**Signal1 + ECE**	0.802 (0,748–0,864)	0.781 (0.724–0.843)	0.776 (0.712–0.845)	0.762 (0.700–0.834)
**Signal1 + Vmax**	0.797 (0,741–0,854)	0.763 (0.701–0.823)	0.790 (0.729–0.856)	0.761 (0.705–0.832)
**Signal1 + dPSA**	0.800 (0,745–0,858)	0.761 (0.702–0.826)	0.780 (0.718–0.851)	0.746 (0.681–0.820)
**Signal1 + Shape + ECE**	0.785 (0,735–0,855)	0.767 (0.708–0.836)	0.758 (0.702–0.840)	0.741 (0.689–0.824)
**Signal1 + Shape + Vmax**	0.784 (0,734–0,849)	0.754 (0.697–0.819)	0.775 (0.722–0.851)	0.747 (0.691–0.828)
**Signal1 + Shape + dPSA**	0.789 (0,738–0,855)	0.756 (0.698–0.829)	0.766 (0.711–0.845)	0.735 (0.678–0.812)
**Signal1 + ECE + Vmax**	0.797 (0,746–0,864)	0.773 (0.714–0.839)	0.777 (0.720–0.850)	0.765 (0.712–0.838)
**Signal1 + ECE + dPSA**	0.805 (0,757–0,866)	0.785 (0.735–0.847)	0.777 (0.719–0.847)	0.765 (0.710–0.834)
**Signal1 + Vmax + dPSA**	0.795 (0,744–0,855)	0.764 (0.708–0.827)	0.790 (0.731–0.857)	0.763 (0.705–0.832)
**Signal1 + Shape + ECE + Vmax**	0.780 (0,733–0,851)	0.762 (0.705–0.832)	0.762 (0.710–0.845)	0.748 (0.693–0.828)
**Signal1 + Shape + ECE + dPSA**	0.789 (0,745–0,858)	0.772 (0.714–0.841)	0.759 (0.705–0.842)	0.746 (0.696–0.825)
**Signal1 + Shape + Vmax + dPSA**	0.785 (0,736–0,849)	0.757 (0.698–0.826)	0.776 (0.720–0.851)	0.748 (0.695–0.824)
**Signal1 + ECE + Vmax + dPSA**	0.800 (0,752–0,861)	0.779 (0.725–0.842)	0.779 (0.727–0.850)	0.764 (0.715–0.834)
**Signal1 + Shape + ECE + Vmax + dPSA**	0.785 (0,738–0,855)	0.767 (0.712–0.838)	0.763 (0.710–0.843)	0.747 (0.697–0.828)

DCE: dynamic contrast-enhanced imaging; AUC: area under the receiver operating characteristic curvewith optimism correction; 95%CI: 95% confidence intervals.

For each reader, the models achieving the highest AUC value were highlighted in green. The models achieving an AUC value that was inferior to the highest AUC value by 0.01 or less were highlighted in yellow.

For all models, AUC values were higher when DCE imaging was used, with a maximal ΔAUC of +0.039 for both readers.

For R1, the Signal1a+ECE+dPSA model obtained the highest AUC (0.805 [95%CI: 0.757–0.866]). It performed better with use of DCE imaging, but the difference was not statistically significant (p = 0.104). As compared to Signal 1a model, Signal1a+ECE+dPSA model achieved a ΔAUC of +0.011, but the difference was not significant (p = 0.167).

For R2, the Signal1a+Vmax+dPSA model obtained the highest AUC (0.790 [95%CI: 0.731–0.857]). It performed better with use of DCE imaging, but the difference was not statistically significant (p = 0.104). As compared to Signal 1a model, Signal1a+Vmax+dPSA model achieved a ΔAUC of +0.021, but the difference was not significant (p = 0.215).

### Comparison of the best multivariable models to the Likert scores in characterizing csPCa-A in PZ

The AUC of the Likert score was 0.844 (95%CI: 0.806–0.877) for R1 and 0.829 (95%CI: 0.791–0.868) for R2. The ΔAUC with the best multivariable model (Signal1a+ECE+dPSA for R1 and Signal1a+Vmax+dPSA for R2) did not reach statistical significance (p = 0.121 for R1, and p = 0.121 for R2).

### Assessment of single variables and multivariable models in characterizing csPCa-B in PZ

Table 5 shows the AUC values achieved by the 9 variables and the different multivariable models developed in the previous steps in characterizing csPCa-B.

**Table 5:** Performances of multivariable models in characterizing csPCa-B in PZ.

	AUC (95%CI)–Reader 1		AUC (95%CI)–Reader 2	
	With DCE	Without DCE	With DCE	Without DCE
**ST2**	0.692 (0.604–0.767)		0.616 (0.554–0.685)	
**SDWI**	0.734 (0.658–0.816)		0.708 (0.647–0.769)	
**SDCE**	0.726 (0.655–0.793)		0.675 (0.604–0.746)	
**S_max**	0.808 (0.741–0.871)	0.767 (0.692–0.837)	0.777 (0.711–0.845)	0.726 (0.660–0.794)
**S_min**	0.604 (0.556–0.652)		0.551 (0.519–0.581)	
**Shape**	0.712 (0.633–0.784)		0.696 (0.619–0.784)	
**ECE**	0.732 (0.659–0.808)		0.720 (0.638–0.801)	
**Vmax**	0.718 (0.643–0.786)		0.695 (0.613–0.774)	
**dPSA**	0.610 (0.447–0.694)		0.623 (0.455–0.707)	
**Signal1**	0.809 (0.741–0.876)	0.764 (0.687–0.838)	0.775 (0.711–0.847)	0.721 (0.658–0.793)
**Signal2**	0.734 (0.665–0.835)	0.739 (0.663–0.827)	0.787 (0.720–0.855)	0.747 (0.687–0.810)
**Signal3**	0.785 (0.720–0.874)	0.754 (0.683–0.841)	0.774 (0.703–0.851)	0.730 (0.664–0.805)
**Signal1 + Shape**	0.803 (0.738–0.878)	0.775 (0.702–0.853)	0.786 (0.718–0.870)	0.760 (0.690–0.844)
**Signal1 + ECE**	0.801 (0.737–0.877)	0.776 (0.705–0.854)	0.787 (0.715–0.866)	0.780 (0.715–0.855)
**Signal1 + Vmax**	0.821 (0.757–0.891)	0.795 (0.721–0.872)	0.813 (0.746–0.882)	0.790 (0.723–0.865)
**Signal1 + dPSA**	0.820 (0.761–0.885)	0.785 (0.705–0.859)	0.791 (0.723–0.869)	0.759 (0.687–0.839)
**Signal1 + Shape + ECE**	0.789 (0.728–0.872)	0.772 (0.694–0.854)	0.777 (0.707–0.866)	0.764 (0.703–0.850)
**Signal1 + Shape + Vmax**	0.813 (0.751–0.886)	0.795 (0.724–0.874)	0.798 (0.727–0.880)	0.777 (0.709–0.866)
**Signal1 + Shape + dPSA**	0.811 (0.753–0.883)	0.786 (0.714–0.865)	0.793 (0.723–0.875)	0.773 (0.704–0.856)
**Signal1 + ECE + Vmax**	0.808 (0.748–0.884)	0.786 (0.714–0.865)	0.799 (0.730–0.871)	0.784 (0.721–0.864)
**Signal1 + ECE + dPSA**	0.811 (0.755–0.881)	0.785 (0.717–0.861)	0.791 (0.725–0.868)	0.783 (0.724–0.863)
**Signal1 + Vmax + dPSA**	0.823 (0.760–0.893)	0.797 (0.722–0.875)	0.809 (0.744–0.884)	0.788 (0.722–0.869)
**Signal1 + Shape + ECE + Vmax**	0.799 (0.740–0.880)	0.782 (0.715–0.868)	0.780 (0.711–0.868)	0.767 (0.703–0.853)
**Signal1 + Shape + ECE + dPSA**	0.799 (0.744–0.879)	0.777 (0.712–0.860)	0.779 (0.714–0.867)	0.773 (0.710–0.857)
**Signal1 + Shape + Vmax + dPSA**	0.815 (0.755–0.888)	0.796 (0.732–0.878)	0.795 (0.731–0.879)	0.777 (0.711–0.865)
**Signal1 + ECE + Vmax + dPSA**	0.811 (0.754–0.887)	0.787 (0.718–0.867)	0.798 (0.734–0.874)	0.783 (0.726–0.868)
**Signal1 + Shape + ECE + Vmax + dPSA**	0.801 (0.747–0.882)	0.782 (0.721–0.872)	0.778 (0.714–0.869)	0.768 (0.705–0.860)

DCE: dynamic contrast-enhanced imaging; AUC: area under the receiver operating characteristic curve with optimism correction; 95%CI: 95% confidence intervals.

For each reader, the models achieving the highest AUC value were highlighted in green. The models achieving an AUC value that was inferior to the highest AUC value by 0.01 or less were highlighted in yellow.

When DCE imaging was used, the addition of other variables to the Signal1a model tended to increase the AUC, with a maximal ΔAUC of +0.014 for R1 and +0.038 for R2. When DCE was not used, all multivariable models performed better than Signal1b model with a maximum ΔAUC of +0.033 for R1 and +0.069 for R2.

For all models, AUC values were higher when DCE imaging was used, with a maximal ΔAUC of +0.035 for R1 and +0.032 for R2.

The highest AUC values were obtained by the Signal1a+Vmax+dPSA model for R1 (0.823 [95%CI: 0.760–0.893]) and by the Signal1a+Vmax for R2 (0.813 [95%CI: 0.746–0.882]).

The AUC of the Likert score was 0.841 (95%CI: 0.799–0.876) for R1 and 0.790 (95%CI: 0.742–0.841) for R2. The ΔAUC between the AUC of the best multivariable model and the Likert score was not significant, neither for R1 (p = 0.52) nor for R2 (p = 0.12).

### Assessment of single variables and multivariable models in characterizing csPCa-C in PZ

When DCE imaging was used, the addition of other variables to the Signal 1a model tended to increase the AUC, with a maximal ΔAUC of +0.031 for R1 and +0.076 for R2 (Table 6). When DCE was not used, all multivariable models performed better than Signal1b model with a maximum ΔAUC of +0.065 for R1 and +0.133 for R2.

**Table 6:** Performances of multivariable models in characterizing csPCa-C in PZ.

	AUC (95%CI)–Reader 1		AUC (95%CI)–Reader 1	
	With DCE	Without DCE	With DCE	Without DCE
**ST2**	0.734 (0.662–0.794)		0.628 (0.560–0.693)	
**SDWI**	0.751 (0.670–0.819)		0.678 (0.617–0.742)	
**SDCE**	0.714 (0.640–0.791)		0.660 (0.584–0.735)	
**S_max**	0.793 (0.713–0.863)	0.752 (0.679–0.823)	0.749 (0.674–0.823)	0.706 (0.628–0.781)
**S_min**	0.624 (0.583–0.666)		0.562 (0.537–0.587)	
**Shape**	0.685 (0.611–0.770)		0.685 (0.596–0.779)	
**ECE**	0.809 (0.727–0.880)		0.824 (0.757–0.892)	
**Vmax**	0.752 (0.680–0.830)		0.750 (0.670–0.828)	
**dPSA**	0.594 (0.448–0.675)		0.614 (0.477–0.698)	
**Signal1**	0.809 (0.731–0.875)	0.768 (0.699–0.837)	0.763 (0.690–0.829)	0.710 (0.636–0.783)
**Signal2**	0.769 (0.694–0.842)	0.766 (0.680–0.840)	0.762 (0.692–0.829)	0.709 (0.638–0.785)
**Signal3**	0.802 (0.739–0.878)	0.782 (0.716–0.851)	0.751 (0.685–0.831)	0.706 (0.635–0.783)
**Signal1 + Shape**	0.798 (0.719–0.875)	0.770 (0.690–0.851)	0.770 (0.703–0.850)	0.729 (0.659–0.821)
**Signal1 + ECE**	0.836 (0.768–0.903)	0.825 (0.766–0.898)	0.835 (0.776–0.894)	0.833 (0.774–0.898)
**Signal1 + Vmax**	0.831 (0.763–0.896)	0.806 (0.742–0.880)	0.818 (0.757–0.886)	0.804 (0.732–0.877)
**Signal1 + dPSA**	0.816 (0.748–0.880)	0.784 (0.713–0.855)	0.780 (0.710–0.854)	0.742 (0.664–0.824)
**Signal + Shape + ECE**	0.819 (0.747–0.895)	0.814 (0.749–0.887)	0.816 (0.756–0.885)	0.814 (0.754–0.886)
**Signal1 + Shape + Vmax**	0.820 (0.752–0.890)	0.800 (0.737–0.877)	0.806 (0.748–0.881)	0.788 (0.723–0.867)
**Signal1 + Shape + dPSA**	0.801 (0.730–0.875)	0.780 (0.708–0.858)	0.775 (0.711–0.859)	0.742 (0.673–0.834)
**Signal1 + ECE + Vmax**	0.839 (0.774–0.906)	0.829 (0.771–0.899)	**0.839 (0.784–0.902)**	**0.843 (0.781–0.907)**
**Signal1 + ECE + dPSA**	0.840 (0.774–0.907)	0.833 (0.776–0.902)	0.836 (0.779–0.897)	0.835 (0.777–0.901)
**Signal1 + Vmax + dPSA**	0.829 (0.764–0.895)	0.806 (0.743–0.881)	0.816 (0.757–0.887)	0.802 (0.733–0.874)
**Signal1 + Shape + ECE + Vmax**	0.825 (0.757–0.902)	0.818 (0.760–0.893)	0.824 (0.770–0.893)	0.823 (0.763–0.897)
**Signal1 + Shape + ECE + dPSA**	0.820 (0.754–0.899)	0.817 (0.757–0.894)	0.817 (0.755–0.888)	0.815 (0.758–0.887)
**Signal1 + Shape + Vmax + dPSA**	0.817 (0.752–0.891)	0.801 (0.736–0.881)	0.804 (0.746–0.880)	0.786 (0.723–0.868)
**Signal1 + ECE + Vmax + dPSA**	0.839 (0.775–0.905)	0.833 (0.778–0.902)	**0.838 (0.786–0.904)**	**0.839 (0.779–0.905)**
**Signal1 + Shape + ECE + Vmax + dPSA**	0.823 (0.755–0.902)	0.819 (0.763–0.895)	0.823 (0.767–0.897)	0.822 (0.763–0.896)

DCE: dynamic contrast-enhanced imaging; AUC: area under the receiver operating characteristic curve with optimism correction; 95%CI: 95% confidence intervals.

For each reader, the models achieving the highest AUC value were highlighted in green. The models achieving an AUC value that was inferior to the highest AUC value by 0.01 or less were highlighted in yellow.

Bold characters indicate models that performed better without DCE imaging.

AUC values tended to be higher when DCE imaging was used, with a maximal ΔAUC of +0.032 for R1 and +0.041 for R2. However, two models performed better without DCE imaging for reader 2, including the model providing the best AUC value.

The highest AUC values were obtained by the Signal1a+ECE+dPSA model for R1 (0.840 [95%CI: 0.777–0.907]) and by the Signal1b+ECE+Vmax for R2 (0.843 [95%CI: 0.781–0.907]).

The AUC of the Likert score was 0.849 (95%CI: 0.811–0.884) for R1 and 0.808 (95%CI: 0.764–0.845) for R2. The ΔAUC between the AUC of the best multivariable model and the Likert score was not significant for R1 (p = 0.49) but was significant for R2 (p = 0.006).

### Evolution of diagnostic performances of models and Likert score as a function of definition of csPCa

Table 7 summarizes the changes in the model performances with changes in csPCa definition. When moving from definition csPCa-A to definition csPCA-C, the ΔAUC between the Signal 1 model and the best multivariate models tended to increase, and the ΔAUC between models with and without DCE tended to decrease. These trends were more pronounced for R2 than for R1.

**Table 7:** Evolution of diagnostic performances of models and Likert scores in PZ as a function of csPCa definition.

Reader		Signal 1a model	Best multivariate model		Likert score	Signal 1a vs multivariable models	Signal 1b vs multivariable models	Multivariable models with vs without DCE
		AUC (95%CI)	Type	AUC (95%CI)	AUC (95%CI)	Median ΔAUC^(1)^ [IQR]	Median ΔAUC^(1)^ [IQR]	Median ΔAUC^(2)^ [IQR]
**R1**	**csPCa-A**	0.794	Signal1+ECE+dPSA	0.805	0.844	-0.005	0.016	0.024
		(0.734–0.851)		(0.757–0.866)	(0.806–0.877)	[-0.009; 0.005]	[0.011; 0.025]	[0.019 ; 0.032]
	**csPCa-B**	0.809	Signal 1 + Vmax + dPSA	0.823	0.841	0.002	0.021	0.024
		(0.741–0.876)		(0.760–0.893)	(0.799–0.876)	[-0.008 ; 0.005]	[0.016 ; 0.027]	[0.019 ; 0.026]
	**csPCa-C**	0.809	Signal 1 + ECE + dPSA	0.840	0.849	0.014	0.046	0.011
		(0.731–0.875)		(0.774–0.907)	(0.811–0.884)	[0.009 ; 0.025]	[0.033 ; 0.054]	[0.007 ; 0.022]
**R2**	**csPCa-A**	0.769	Signal1+Vmax+dPSA	0.790	0.829	0.007	0.023	0.017
		(0.698–0.835)		(0.731–0.857)	(0.791–0.868)	[-0.007; 0.009]	[0.021; 0.038]	[0.014; 0.029]
	**csPCa-B**	0.775	Signal 1 + Vmax	0.813	0.790	0.016	0.056	0.015
		(0.711–0.847)		(0.746–0.882)	(0.742–0.841)	[0.008 ; 0.023]	[0.047 ; 0.062]	[0.012 ; 0.021]
	**csPCa-C**	0.763	Signal 1 + ECE + Vmax	0.843	0.808	0.054	0.104	0.002
		(0.690–0.829)		(0.781–0.907)	(0.764–0.845)	[0.042 ; 0.067]	[0.077 ; 0.118]	[0.001 ; 0.018]

AUC: area under the receiver operating characteristic curve with optimism correction; ΔAUC: difference between two AUC values; 95%CI: 95% confidence intervals; R1: reader 1; R2: reader 2.

(1) A positive difference indicates a better performance of the multivariable models.

(2) A positive difference indicates a better performance of the model using DCE imaging.

## Discussion

The purpose of this study was not to build a new scoring system that could compete with existing ones, but rather to define the most informative MR features in characterizing csPCa in PZ, and to assess the relative diagnostic weight of these features, and how they could be optimally combined. Answering these questions is indeed mandatory if one wants to improve existing scores in the future.

To achieve this purpose, we used a radiologic-pathologic database containing a detailed description of sequence-specific features of all lesions visible at pre-operative prostate multiparametric MR imaging. This description was made prospectively by two independent readers and was then compared to prostatectomy specimens findings used as reference.

We used several definitions for csPCa since there is currently no consensus on this matter. As a primary objective, we defined csPCa as cancers with a Gleason score ≥7 because the metastatic and lethal potential of Gleason 6 cancers is low [40], and because this definition is commonly used [41]. Using this definition, we built the best multivariate models through a stepwise approach. Then, the models were assessed using more stringent definitions for csPCa.

Unsurprisingly, S_DW gave consistently better results than S_T2, and S_DCE for both readers, whatever the definition used for csPCa. This confirms that DW imaging is the most informative pulse sequence in PZ. Nonetheless, combining the results of the three pulse sequences into S_Max resulted in a substantial improvement in the diagnostic performance for both readers. This is in line with the good results obtained at the National Institute of Health (NIH) with an in-house score taking into account only the number of positive pulse sequences [16, 42], and points out that signal abnormalities remain the most informative features and should play a central role in any scoring system.

The Signal1-3 models consisted in three different combinations of the signal-based variables. They provided only marginal improvement as compared to S_Max. Particularly, the Signal2 model, that was an attempt to increase the diagnostic weight of DW imaging among the signal-based variables, failed to improve the characterization of csPCa. Thus, although DW imaging is the most informative pulse sequence in PZ, its optimal combination with the other pulse sequences remains to be defined.

Because the Signal1 model tended to give the best results, it was selected for the next step that assessed the added value of variables that were not related to signal abnormalities. Three variables (dPSA, ECE and V_Max) showed consistent added value and all best multivariable models included at least one of them. Their added value over signal-based variables (i.e. the ΔAUC between the best multivariable model and the Signal 1 model) tended to increase when DCE imaging was not used and when more stringent definitions of csPCa were used. Unsurprisingly, the diagnostic weight of ECE increased when the definition of csPCa included extraprostatic extension (csPCa-C), and the diagnostic weight of V_Max increased when the definition of csPCa was restricted to more aggressive tumors (csPCa-B instead of csPCa-A). Thus, an international consensus on the definition of csPCa is becoming crucial, since this will impact the scoring system to be used on multiparametric MR imaging. Interestingly, good results have recently been reported with a refinement of the NIH score combining ECE features and the number of positive pulse sequences [28]. Our results are in line and suggest that ECE features not only assess prostate cancer extracapsular extension, but also help characterizing the nature of the lesion. dPSA was the only non-MR variable included in this study. The fact that it consistently provided independent information to MR features is in line with a recent study that found that combining the PIRADS v2 score with dPSA improved prostate lesion characterization [43]. If the aim of scoring systems is to assess the likelihood of presence of csPCa, it might therefore be necessary to associate MR features and clinical or biochemical features in the future.

Taking into account the shape of the lesions decreased the performance of almost all models. This strongly suggests that shape is not a good predictor of csPCa, even if the PIRADS v2 and other scoring systems [15, 17] use it to characterize focal lesions in PZ. The poor diagnostic value of shape had already been found in another study [14] and may be due to the fact that it remains a very subjective feature.

There is currently a controversy about the added value of DCE imaging, as compared to T2W and DW imaging. DCE imaging may indeed help detect small cancers, but may also increase the number of false positive findings [23, 44–47]. Several groups failed to find clear added value for DCE imaging when MR images were interpreted visually or using scoring systems [25, 48–50]. Nonetheless, in most quantitative studies aimed at characterizing prostate lesions [34, 51–54], DCE-derived parameters were part of the best final models. Our study is in line with these quantitative studies. When we removed DCE findings, we observed a systematic decrease in the diagnostic performances of nearly all models, for both readers, suggesting that DCE imaging does provide information. The best way to incorporate this information into a scoring system remains to be defined. Interestingly, the added value of DCE imaging tended to decrease for both readers when more stringent definitions of csPCa were used.

In the last part of our study, we compared the best models to the Likert score prospectively assigned to each lesion. For the most experienced reader, the Likert score constantly outperformed the best model, even if the difference was never statistically significant and tended to decrease as more stringent definitions of csPCa were used. For the least experienced reader, however, the best model outperformed the Likert score for csPCa-B and csPCa-C, and the difference was statistically significant for csPCa-C. Most of the image features we used were subjective and this may be seen as a contradiction with our initial goal to obtain more objective scoring systems. Unless an entirely quantitative approach is used for all pulse sequences, subjective assessment of images remains unavoidable. Existing scoring systems share the same limitation, as shown by the PIRADS v2 score that distinguishes indistinct hypointense (score 2), mildly/moderately hypointense (score 3) or markedly hypointense (scores 4–5) lesions on ADC maps. However, our results suggest that breaking down the diagnostic process into separate features, and combining these features into predefined models may help less experienced readers better characterise MR lesions.

Our study has some limitations.The description of the sequence-specific features was done prospectively. Although this could be seen as a strength of the study, it also induced two limitations. First, we were not able to compare the best models with the PIRADS v2 score that was not launched at the start of the study. However, our main purpose was not to compare our models to existing scores, but to understand the features’ relative weight in characterizing csPCa. Second, the features noted in the database for TZ lesions were mostly based on signal abnormalities. Features as homogeneous pattern, presence of a capsule, apical or anterior location, that are now known as major predictors of malignancy in TZ [55, 56] were not prospectively recorded. As a result we chose not to use the database for assessing diagnostic models in TZ. Another limitation is due to the fact that both readers were from the same institution and may have characterized prostate lesions in a similar way. The best multivariable models may have been different with readers from other institutions. Finally, our study mixed patients imaged at different field strengths with varying protocols. Although this resulted in a heterogeneous population, it may better reflect daily routine population.

## Conclusion

The number of pulse sequences showing marked signal abnormality for a given lesion (S_max) was one the most informative variable for both readers, whatever the definition used for csPCa. A moderate improvement could be obtained by taking into account, in addition to S_max, the number of negative pulse sequences, the presence of extracapsular extension features, the volume of the lesion and the PSA density. The added value of the three latter variables depended on the definition used for csPCa and tended to increase when more stringent definitions were used. Removing DCE findings decreased performance in nearly all models, but the difference decreased when more stringent definitions were used for csPCa. Finally the Likert score outperformed the best multivariable models for the most experienced reader whatever the definition used for csPCa. For the other reader, the multivariable models outperformed the Likert score for csPCa-B and csPCa-C definitions, and the difference was significant for csPCa-C. This suggests that scoring systems based on semi-objective variables may help less-experienced radiologists.

## Supporting information

S1 Table MR imaging parameters. TR: Repetition Time; TE: Echo time; PPA: pelvic phased array; T2w: T2 weighted imaging; Dw: Diffusion weighted imaging; DCE: dynamic contrast enhanced imaging.
(DOCX)

S2 Table Distribution of MR-derived individual variables according to the nature of the lesions and according to the reader. Sd: standard deviation; IQR: interquartile range.
(DOCX)

S1 Appendix Method for correcting optimism in estimating the area under the receiver operating characteristic curve. (DOCX)
