# Supplementary material for: Diagnostic value and relative weight of sequence-specific magnetic resonance features in characterizing clinically significant prostate cancers
Source: PLoS One. 2017 Jun 9;12(6):e0178901. doi: 10.1371/journal.pone.0178901 (PMC5466299; doi:10.1371/journal.pone.0178901)
Supplement: S1 Table — TR: Repetition Time; TE: Echo time; PPA: pelvic phased array; T2w: T2 weighted imaging; Dw: Diffusion weighted imaging; DCE: dynamic contrast enhanced imaging. (DOCX) [file pone.0178901.s001.docx]

|  |  | Coil type | Sequence | TR(ms) | TE (ms) | Field of view (mm) | Matrix | Section thickness (mm) | Flip Angle (degrees) | b-Value | Acquisition number | Temporal resolution (s) |
| --- | --- | --- | --- | --- | --- | --- | --- | --- | --- | --- | --- | --- |
| A | 1,5T Siemens Medical Systems | PPA coil only | T2w | 7750 | 109 | 200/200 | 256/184 | 3 | 180 |  |  |  |
|  |  |  | Dw | 4800 | 90 | 300/300 | 123/83 | 3 | 90 | 0-600 |  |  |
|  |  |  | DCE | 5.4 | 2.7 | 240/240 | 256/135 | 3 | 10 |  | 12 | 15 |
| B | 3T General Electric Medical Systems | PPA coil only | T2w | 5000 | 104 | 220/220 | 384/256 | 3 | 90 |  |  |  |
|  |  |  | Dw | 5000 | 90 | 380/380 | 128/128 | 3 | 90 | 0-2000 |  |  |
|  |  |  | DCE | 3.9 | 1.7 | 240/192 | 180/160 | 3 | 12 |  | 32 | 7 |
| C | 3T Philips Medical Systems | Combined PPA and endorectal coils | T2w | 5021 | 120 | 180/180 | 344/255 | 3 | 90 |  |  |  |
|  |  |  | Dw | 3925 | 70 | 180/180 | 116/103 | 3 | 90 | 0-800-2000 |  |  |
|  |  |  | DCE | 4 | 2.3 | 180/180 | 100/100 | 3 | 8 |  | 50 | 5 |

**Supporting Table 1: MR imaging parameters**

TR : Repetition Time ; TE : Echo time ; PPA : pelvic phased array; T2w : T2 weighted imaging ; Dw : Diffusion weighted imaging ; DCE : dynamic contrast enhanced imaging.
