# Supplementary material for: Diagnostic value and relative weight of sequence-specific magnetic resonance features in characterizing clinically significant prostate cancers
Source: PLoS One. 2017 Jun 9;12(6):e0178901. doi: 10.1371/journal.pone.0178901 (PMC5466299; doi:10.1371/journal.pone.0178901)
Supplement: S2 Table — Sd: standard deviation; IQR: interquartile range. (DOCX) [file pone.0178901.s002.docx]

| Radiologic features | | Reader 1 | | Reader 2 | |
| --- | --- | --- | --- | --- | --- |
|  | | **Benign/Gleason ≤6 (n=268)** | **Gleason ≥7 (n=204)** | **Benign/Gleason ≤6 (n=198)** | **Gleason ≥7 (n=191)** |
| S_T2 | Not visible | 15 (5.60 %) | 5 (2.45 %) | 4 (2.0 %) | 0 (0.00 %) |
|  | Mild | 104 (38.8 %) | 29 (14.2 %) | 18 (9.09 %) | 7 (3.66 %) |
|  | Moderate | 113 (42.2 %) | 73 (35.8 %) | 80 (40.4 %) | 40 (20.9 %) |
|  | Marked | 36 (13.4 %) | 97 (45.6 %) | 96 (48.5 %) | 144 (75.4 %) |
| S_DW | Not visible | 55 (20.5 %) | 16 (7.84 %) | 7 (3.54 %) | 2 (1.05 %) |
|  | Mild | 108 (40.3 %) | 35 (17.2 %) | 30 (15.2 %) | 8 (4.19 %) |
|  | Moderate | 73 (26.2 %) | 48 (23.5 %) | 87 (43.9 %) | 31 (16.2 %) |
|  | Marked | 32 (11.9 %) | 105 (51.5 %) | 74 (37.3 %) | 150 (78.5 %) |
| S_DCE | Not visible | 41 (15.3 %) | 10 (4.9 %) | 22 (11.1 %) | 3 (1.57 %) |
|  | Mild | 80 (29.9 %) | 37 (18.1 %) | 35 (17.7 %) | 13 (6.81 %) |
|  | Moderate | 99 (36.9 %) | 45 (22.1 %) | 68 (34.4 %) | 45 (23.6 %) |
|  | Marked | 48 (17.9 %) | 112 (54.9 %) | 73 (36.9 %) | 130 (68.1 %) |
| S_Min | 0 | 176 (65.7%) | 177 (86.8%) | 166 (83.8%) | 186 (97.4%) |
|  | ≥1 | 92 (34.3%) | 27 (13.2%) | 32 (16.2%) | 5 (2.6%) |
| S_Max | 0 | 176 (65.7%) | 42 (20.6%) | 49 (24.7%) | 13 (6.8%) |
|  | 1 | 72 (26.9%) | 62 (30.4%) | 77 (38.9%) | 26 (13.6%) |
|  | 2 | 16 (6%) | 48 (23.5%) | 51 (25.8%) | 58 (30.4%) |
|  | 3 | 4 (1.5%) | 52 (25.5%) | 21 (10.6%) | 94 (49.2%) |
| Shape | Ill-defined area, or linear perpendicular to the capsule | 92 (34.3 %) | 35 (17.2 %) | 52 (26.3 %) | 20 (10.5 %) |
|  | Linear parallel to the capsule | 32 (11.9 %) | 15 (7.35 %) | 9 (4.55 %) | 6 (3.14 %) |
|  | Triangular | 40 (14.9 %) | 19 (9.31 %) | 60 (30.3 %) | 38 (19.9 %) |
|  | Nodular without mass effect | 102 (38.1 %) | 102 (50 %) | 64 (32.3 %) | 74 (38.7 %) |
|  | Nodular with mass effect | 2 (0.746 %) | 33 (16.2 %) | 13 (6.57 %) | 53 (27.7 %) |
| ECE | 1 | 219 (81.7%) | 80 (39.2%) | 95 (48%) | 37 (19.4%) |
|  | 2 | 20 (7.5%) | 29 (14.2%) | 66 (33.3%) | 60 (31.4%) |
|  | 3 | 25 (9.3%) | 28 (13.7%) | 26 (13.1%) | 33 (17.3%) |
|  | 4 | 3 (1.1%) | 40 (19.6%) | 5 (2.5%) | 31 (16.2%) |
|  | 5 | 1 (0.4%) | 27 (13.2%) | 6 (3%) | 30 (15.7%) |
| V_max (3) | Median (Sd) | 0.69 (1.15) | 1.38 (3.47) | 0.75 (1.14) | 1.52 (2.55) |
|  | IQR | 0.2475-1.51 | 0.52-2.83 | 0.385-1.783 | 0.64-3.045 |
|  | Min-Max | 0.02-6.17 | 0.05-32.7 | 0.03-4.7 | 0.05-14.62 |
| Likert | 2 | 73 (27.2%) | 8 (3.9%) | 17 (8.6%) | 2 (1%) |
|  | 3 | 109 (40.7%) | 24 (11.8%) | 94 (47.5%) | 14 (7.3%) |
|  | 4 | 76 (28.4%) | 58 (28.4%) | 66 (33.3%) | 54 (28.3%) |
|  | 5 | 10 (3.7%) | 114 (55.9%) | 21 (10.6%) | 121 (63.4%) |

**Supporting Table 2: Distribution of MR-derived individual variables according to the nature of the lesions and according to the reader.**

Sd: standard deviation; IQR: interquartile range.
