# Supplementary material for: Diagnostic value and relative weight of sequence-specific magnetic resonance features in characterizing clinically significant prostate cancers
Source: PLoS One. 2017 Jun 9;12(6):e0178901. doi: 10.1371/journal.pone.0178901 (PMC5466299; doi:10.1371/journal.pone.0178901)
Supplement: S1 Appendix — (DOCX) [file pone.0178901.s003.docx]

**S1 - Appendix**

We present here the method used to estimate the corrected AUC (c.AUC) for each combination of variables. For a given combination of variables, a logistic regression was used on the original dataset to predict the probability that the lesion correspo,ded to a Gleason ≥7 cancer, in order to build the ROC curve and to estimate the non-corrected AUC.

The aim of the following method was to estimate the optimism of the non-corrected AUC and to subtract it from the non-corrected AUC to obtain an unbiased estimate (c.AUC).

For $i \epsilon\{1,\ldots,999\}$,

1. A set of patients was resampled with replacement from the original dataset e l to obtain a boostrap dataset that included all the lesions of the sampled patients. The bootstrap dataset is called Xboot_i
2. A logistic regression was carried out on the bootstrap dataset, including the same combination of variables than the model applied on the original dataset. This model is called *model_boot_i.*
3. A ROC curve was built using the probabilities of Gleason ≥7 cancer predicted by the *model_boot_i* for the lesions of the Xboot_i dataset and the corresponding AUC was estimated. This AUC is called AUC_i.

A *crossed estimate of the AUC* was obtained (using the probabilities of Gleason ≥7 cancer predicted by the *model_boot_i* for the lesions not included in the bootstrap sample (namely X\Xboot_i). This AUC is called crAUC_i.

1. The value o_i = AUC_i – crAUC_i is an estimate of the optimism.

The corrected AUC was obtained by subtracting the mean of the 999 o_i from the non-corrected AUC.
c.AUC = AUC – mean(o_i)

The crossed estimates of AUC (crAUC_i) are estimates of the corrected AUC. The confidence interval of the corrected AUC was built using the 2.5th and 97.5th empirical quantiles of the distribution of the crossed estimates.

The distribution of the differences between the crossed estimates of AUC (cr.AUC_i) of two models applied to the same bootstrap samples was used to estimate the standard error of the difference between the two corresponding corrected AUCs in order to test this difference. The 2.5th and 97.5th empirical quantiles of the distribution of the differences were also used to build the confidence interval of the difference between two corrected AUCs.
